# Supplementary material for: Consolidated Health Economic Evaluation Reporting Standards 2022 (CHEERS 2022) statement: Updated reporting guidance for health economic evaluations
Source: Health Policy Open. 2022 Jan 5;3:100063. doi: 10.1016/j.hpopen.2021.100063 (PMC10297740; doi:10.1016/j.hpopen.2021.100063)
Supplement: Supplementary data 1 [file mmc1.docx]

**Supplementary Information**

Consolidated Health Economic Evaluation Reporting Standards 2022 (CHEERS 2022) statement: updated reporting guidance for health economic evaluations

Don Husereau1 Michael Drummond,2 Federico Augustovski,3 Esther de Bekker-Grob,4 Andrew H Briggs,5 Chris Carswell,6 Lisa Caulley,7 Nathorn Chaiyakunapruk,8 Dan Greenberg,9 Elizabeth Loder,10 Josephine Mauskopf,11 C Daniel Mullins,12 Stavros Petrou,13 Raoh-Fang Pwu,14 Sophie Staniszewska,15

1. School of Epidemiology and Public Health, University of Ottawa, Ontario, Canada; Institute of Health Economics, Alberta, Canada

2. Centre for Health Economics, University of York, York, UK

3. Health Technology Assessment and Health Economics Department of the Institute for Clinical Effectiveness and Health Policy (IECS-CONICET), Buenos Aires; University of Buenos Aires, Buenos Aires; CONICET (National Scientific and Technical Research Council), Buenos Aires, Argentina

4. Erasmus School of Health Policy & Management, Erasmus University Rotterdam, Rotterdam, The Netherlands

5. London School of Hygiene and Tropical Medicine, London, England, UK

6. Adis Journals, Springer Nature, Auckland, New Zealand

7. Department of Otolaryngology - Head & Neck Surgery, University of Ottawa, Ontario, Canada; Clinical Epidemiology Program and Center for Journalology, Ottawa Hospital Research Institute, Ontario, Canada; Department of Epidemiology, Erasmus University Medical Center Rotterdam, Rotterdam, The Netherlands

8. Department of Pharmacotherapy, College of Pharmacy, University of Utah, Salt Lake City, Utah, USA

9. Department of Health Policy and Management, School of Public Health, Faculty of Health Sciences, Ben-Gurion University of the Negev, Be’er-Sheva, Israel

10. Harvard Medical School, Boston, MA, USA; *The BMJ*, London, UK

11. RTI Health Solutions, RTI International, Research Triangle Park, NC, USA

12. School of Pharmacy, University of Maryland Baltimore, Baltimore, MD, USA

13. Nuffield Department of Primary Care Health Sciences, University of Oxford, Oxford, UK

14. National Hepatitis C Program Office, Ministry of Health and Welfare, Taipei City, Taiwan

15. Warwick Research in Nursing, University of Warwick Warwick Medical School, Warwick, UK

Correspondence to: D Husereau donh@donhusereau.com

**Append A: GRIPP2 – Short Form: Public Involvement in CHEERS**

| Aims | 1. To establish an international public reference group to guide public involvement in CHEERS. 2. To embed public involvement at each key stage of the CHEERS checklist update. |
| --- | --- |
| Methods | We were unable to identify specific reporting guidance to guide the reporting of patient and public involvement and engagement (PPIE) in health economic evaluation, although GRIPP2 provides generic guidance on high quality PPIE reporting (Staniszewska et al 2017). Recognising the potential for PPIE in health economics research more generally, we identified the need for CHEERS to include items that enable reporting of PPIE and community engagement in health economic evaluation.  We established a public reference group made up of individuals with an interest in the reporting of health economic evaluation, who have knowledge of research and HTA and have been involved in a range of studies. We purposively selected individuals who would represent a public view, rather than a specific area of patient experience, as we recognised that the discussion about CHEERS items would happen at a macro level, rather than focusing on specific areas of patient experience.  We used a series of meetings or ‘knowledge spaces’ to create opportunities for deliberative dialogue about CHEERS. These meetings included the research team presenting on the background and development of CHEERS. In the first meeting we reviewed the CHEERS items with public contributors commenting on item wording and meaning. Each item was considered separately. The research team then edited the items, drafted new PPIE items and circulated that to the Public Reference group. These items were then discussed at the second meeting, prior to the Delphi exercise, ensuring PPIE was built in early in the process. In meeting three the focus was on reviewing progress, developing ideas for resources to support patient and public in dissemination of CHEERS. The draft paper, the document supporting PPIE in involvement in health economic evaluation and final checklist was sent to the PPIE Reference group for comment and input which was acted on. |
| Results | Each item was reviewed by the Public Reference Group and discussed with some editing to clarify meaning form a public perspective.  The Public reference Group identified the need for additional items to capture any patient and public involvement in a health economic evaluation. After discussion with the Public Reference group and with wider collaborators and following a process of editing and refinement, two key items were included in the checklist. These items are Items 21 and 25.  In addition to discussions about the items, the Public Reference Group also discussed the need to create resources that support patients and public contributors to engage in discussions about health economics. In response to input from the public reference group we developed a guide to support patient and public involvement in health economic evaluation, which the public reference group commented on, resulting in further refinements. For examples, they suggested we include all items rather than a selection of indicative items. This document will be published separately The Group also identified a range of dissemination approaches that would support public awareness and potential use of CHEERS. |
| Discussion | Public involvement in a health economic evaluation is in its infancy but it has the potential to contribute new forms of knowledge, important insights and ultimately enhance the validity, trustworthiness, legitimacy and accountability of health economic evaluation. The CHEERS PPIE items will enable the reporting of patient and public involvement and contribute to a strengthening evidence base underpinning health economic evaluation. |
| Reflections | CHEERS PPIE items will encourage reporting of involvement and engagement when it is carried out but also encourage health economics to consider the potential benefits of involving patients and the public in their work. |

# Appendix B: Protocol for Delphi Process

## Objective

The overarching objective is to produce a minimum number of required reporting elements for health economic evaluations. It should be remembered that CHEERS is about the reporting of economic evaluations, that have been defined as ‘the comparative analysis of alternative courses of action in terms of both their costs and consequences.’ This will be done through capturing and conveying judgment from experts in health economic evaluation and medical journal editing.

## Context and Rationale

Reports of economic evaluations in health care should be clear about why a study was required, how it was conducted (including its data sources and key assumptions), and what its findings and conclusions are. This is critical for it to be understood and useful to policymakers, providers, and patients. Moreover, these stakeholders need to interpret study findings.

Reporting guidelines that consist of a minimum number of required reporting elements have been proposed as a practical method of helping authors, editors, and peer-reviewers produce reports that convey details of a study transparently and consistently. Which reporting elements in any scientific article are minimally required, however, is a matter of subjective opinion. Consensus methods such as a Delphi process[1,2] are useful to capture the minimum number of elements of reporting using a variety of expertise while minimizing the unnecessary influence of dominant experts in a group.

## Methods and timelines

### **Participants**

Participants are expected to have expertise in

1. the conduct, analysis and interpretation of health economic evaluation; or
2. biomedical journal editing and reviewing.

It may be additionally important to invite those who can correctly apply the findings of studies including care providers, patients, members of the public and policy makers.

A purposive sample will be selected by Task Force Members who will individually approach potential participants through email. Potential participants will then have their participation confirmed by an official letter of invitation sent by the ISPOR Staff Liaison / Delphi Administrator.

### **Process**

A modified Delphi process [2,3] will be conducted by a delegated member of the ISPOR CHEERS II Task Force. Participants will be asked to vote on the relevance of candidate reporting elements on a checklist. Reporting elements will be numbered and arranged under conventional ordering of sections for an economic evaluation report (e.g., title, abstract, introduction, methods, etc.).

Each item will have a reporting item description, an associated definition of what is meant by the item and a rationale for inclusion. An example is as follows:

Item: DISCOUNT RATE - Report the choice of discount rate(s) used for costs and outcomes and say why appropriate.

Definition: “Discount rate – an annual rate, described in percentage, used to calculate current values of resources or health outcomes”.

Rationale for inclusion: Rationale for inclusion: the discount rate reflects societal preferences for immediate gains in health or wealth versus gains that occur further into the future. The choice of discount rate affects the “present” value of costs and consequences that occur in the future. It is therefore important that the reader is aware of the discount rate chosen and the reasons for the choice, especially for programmes where the costs and consequences occur over several years.

An email reminder will be sent to participants 7 days and 4 days before the deadline for each round of the survey.


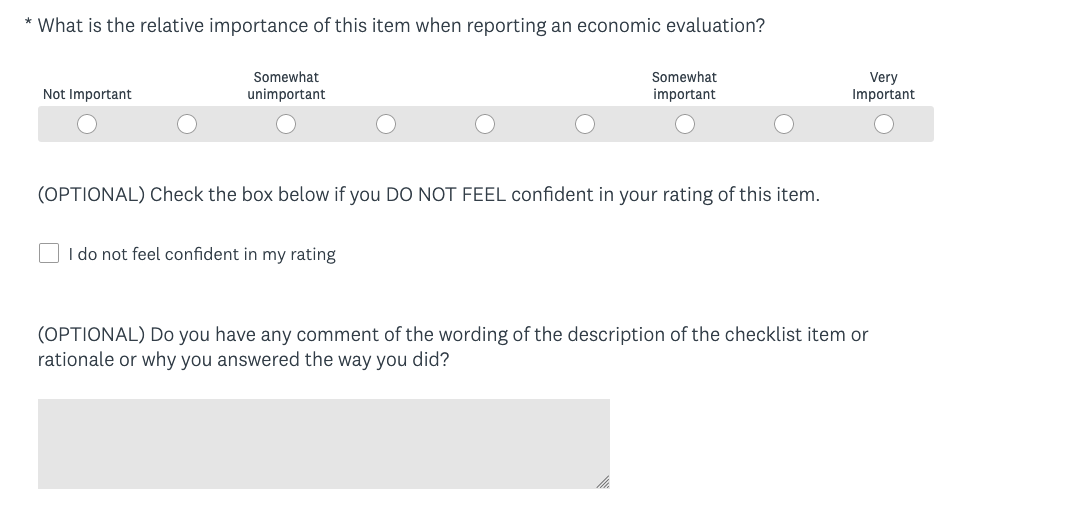


Demographic information including, sex, age, location of work, region of work, and years of experience will also be collected.

Each participant’s responses will be collated, and responses entered onto an Excel spreadsheet. Measures of central tendency and variability (mean and medians, with 95% CI and IQR distributions as well as a maximum-minimum range for each response, weighted by participant confidence) will be calculated. Participants will also have the opportunity to comment on the checklist items or to suggest additional items.

We a priori define a consensus-equivalent threshold of ≥70%, to support inclusion of an item into the second round. An item will be removed if more than 70% of the members score it as < 7 on the scale. Participants will be asked to comment on the wording and response options, and propose new items, if needed. A summary of the rating and feedback on each remaining item will be created.

In the second round, checklist items will be listed in order of importance, using the mean scores and median score, the inter-percentile range (IPR) (30th and 70th), and the inter-percentile range adjusted for symmetry (IPRAS), for each item (_i_) being rated. (Fitch K, Bernstein S, et al.) from round one. Respondents will be asked to revisit their answer in light of these scores and revise. They will be asked to provide a rationale for revising their original estimate including evidence that they think is relevant. Participants will be told that items with a mean score of ≥ 7 will be grouped as ‘Included’ checklist items. Items with a mean score of > 4 and < 7 will be grouped as ‘Possible’ checklist items and might not be included in the final checklist.

Items with a mean score of 1 to 4 will be grouped as a ‘Rejected’ checklist list item. Rejected items will not be included in the final checklist unless they receive much higher scores in round two. Newly introduced items will also be voted upon this round.

If ‘Possible’ items still exist, a third round may be initiated. Again, checklist items will be listed in order of importance, using the mean scores from round one. Respondents will be presented with reasons for high and low estimates of importance. ‘Possible’ items that score do not score higher than 4 will be removed from the checklist. ‘Possibles’ that score above 5 will be included. Participants will then be invited to comment on which arguments they found unconvincing and why.

If required, the Delphi survey will then proceed to a final round, where respondents are presented with scores along with counterarguments against reasons for high and low estimates of importance, particularly for those items scored as ‘Possible’. Participants are asked to create a final revision of their ratings.

In each round, respondents will be given 7 days to provide responses. Reminders will be sent out 3 days and 1 day before the deadline. A week will be then taken to analyze responses and accumulate any late responses. A flowchart of participation including number approached, and subsequently unavailable or non-responsive will be kept. In all analyses, sensitivity will be conducted by respondent self-reported confidence in their response. In some cases, mean values may be taken from those who were most confident, if there is a wide variation in responses reported.

**Appendix Table B1 Process**

| Deliverables / Actions | Description | Date | Output |
| --- | --- | --- | --- |
| Discuss/Finalize Protocol | Committee Members to discuss-suggest additions to protocol at teleconference and subsequent emails | Aug, 2020 | Final Protocol for Delphi |
| Finalize the Panel | Each Committee Member Identifies Individuals with Expertise for Potential Interest | May-June, 2020 | List of 30-40 potential experts sent to Delphi Administrator |
| Gauge interest in participation | TF Member sends email to see if member is interested | Aug, 2020 | List of interested participants |
| Invitation to participate | Administrator sends out official invitation to participate | Aug, 2020 | Contact established with Delphi administrator. Final list of participants |
| Discuss/Finalize Delphi Questionnaire | Draft questionnaire sent to CHEERS TF members for comments | Aug, 2020 | Final Questionnaire |
| 1^st^ Round of Delphi Process | Respondents asked to rate the importance of each element and rate their ability to answer | Sept, 2020 | Complete first round of Delphi process |
| 2^nd^ Round of Delphi Process | Respondents asked to revisit their answer in light of importance and revise it. They will be asked to provide a rationale for revising their original estimate | Sept, 2020 | Complete second round of Delphi process |
| Possible 3^rd^ Round of Delphi Process | Respondents presented with median and IQR along with reasons for high and low estimates of importance. Participants asked to specify which arguments they found unconvincing and why | Oct, 2020 | Complete 3^rd^ Round |
| Possible 4^th^ Round of Delphi Process | Respondents presented with median and IQR along with counterarguments against reasons for high and low estimates of importance. Participants asked to revise | Oct, 2020 | Draft Prepared |
| Acknowledgment | Respondents will be thanked and asked if they wish to be acknowledged in the Task Force publication | Oct, 2020 |  |

### **Invitation Letter for Survey Nominees**

Subject: ISPOR Task Force on Health Economic Evaluation Reporting Guidelines - Interest in Participating in a Delphi Panel survey

[Name],

I am wondering if you would be interested in participating in a Delphi panel survey being conducted by the International Society for Pharmacoeconomics and Outcomes Research (ISPOR).

We are interested in capturing the opinions of 30-40 internationally recognized experts from [biomedical journal editing, health economic evaluation, patient experts, decision makers, industry] to develop a minimum set of standard reporting requirements for health economic evaluations. The minimum set will constitute a ‘checklist’ of reporting requirements.

The Delphi survey will be administered by email and begin in the third week of September and could require 60 minutes of your time in the first round. It is anticipated the survey will be repeated once (and possibly twice) every two to three weeks after this time.

It is hoped that the checklist will be published next year and if you wish, you could be acknowledged as a survey participant. If you are interested in participating in the survey, please let me know by [DATE], and I will pass on your name to ISPOR, who will send you an official invitation shortly.

Cheers,

[Don and Mike]

**References**

1 Moher D, Schulz KF, Simera I, *et al.* Guidance for developers of health research reporting guidelines. *PLoS Med* 2010;**7**:e1000217. doi:10.1371/journal.pmed.1000217

2 Fitch K, editor. *The Rand/UCLA appropriateness method user’s manual*. Santa Monica: : Rand 2001.

3 Hasson F, Keeney S, McKenna H. Research guidelines for the Delphi survey technique. *J Adv Nurs* 2000;**32**:1008–15.
